# Translation and Validation of the Caffeine Expectancy Questionnaire in Brazil (CaffEQ-BR)

**DOI:** 10.3390/nu12082248

**Published:** 2020-07-28

**Authors:** Guilherme Falcão Mendes, Caio Eduardo Gonçalves Reis, Eduardo Yoshio Nakano, Teresa Helena Macedo da Costa, Bryan Saunders, Renata Puppin Zandonadi

**Affiliations:** 1 Department of Nutrition, School of Health Sciences, University of Brasilia (UnB), Campus Darcy Ribeiro, Asa Norte, Brasilia DF 70910-900, Brazil; caioedureis@gmail.com (C.E.G.R.); thmdacosta@gmail.com (T.H.M.d.C.); 2 Department of Statistics, Central Institute of Sciences, University of Brasilia (UnB), Campus Darcy Ribeiro, Asa Norte, Brasilia DF 70910-900, Brazil; eynakano@gmail.com; 3 Applied Physiology and Nutrition Research Group, School of Physical Education and Sport; Rheumatology Division; Faculdade de Medicina FMUSP, University of São Paulo, Sao Paulo 01246-903, Brazil; drbryansaunders@outlook.com; 4 Institute of Orthopaedics and Traumatology, Faculty of Medicine FMUSP, University of São Paulo, Sao Paulo 01246-903, Brazil

**Keywords:** caffeine, subjective, expectancy, instrument, validation, Brazilian, Portuguese

## Abstract

Caffeine is the world’s most commonly used stimulant of the central nervous system. Caffeine is present in coffee and other beverages such as tea, soft drinks, and cocoa-based foods. The caffeine expectancy questionnaire was developed to investigate the effects of caffeine expectations and thus contribute to knowledge about its usage and subjective effects (response expectancies). This study aimed to evaluate caffeine expectation psychometrically in a sample of the Brazilian population. The original version of the “Caffeine Expectancy Questionnaire (CaffEQ)” was translated and validated into Brazilian-Portuguese and adapted to Brazilian culture to be used in the Brazilian adult (19–59 y) population. After the translation and back-translation processes of the original CaffEQ questionnaire, the content and semantic validation were performed by a group of experts. The Brazilian-Portuguese version of the questionnaire consists of 47 items, in seven factors, which assess subjective perceptions about the effects of caffeine. Interobserver reproducibility and internal consistency of the questionnaire were tested with a convenience sample (*n* = 50) of Brazilian adult consumers of caffeine sources, who completed the Brazilian CaffEQ (CaffEQ-BR) on two occasions separated by 24 h. All of the 47 questions were adequate regarding reliability, clarity, and comprehension. Psychometric properties could be replicated consistently. Appropriate internal consistency and validation were confirmed by Cronbach’s alpha (α) 0.948, and an intraclass correlation coefficient of 0.976 was observed. The CaffEQ-BR was applied using a web-based platform to a convenience sample of Brazilian adults from all 27 Brazilian states (*n* = 4202 participants), along with measures of sociodemographic and caffeine consumption data. Factor validity was verified by confirmatory factor analysis. The seven factors presented a good fit for Root Mean Square Error of Approximation—RMSEA = 0.0332 (95% CI: 0.0290–0.0375). By confirming the validity and reliability of CaffEQ-BR, a useful tool is now available to assess caffeine expectations in the Brazilian adult population.

## 1. Introduction

Caffeine (1,3,7-trimethylxanthine) is the most widely consumed psychoactive substance in the world [1,2] with several guidelines addressing the form of use, dosage, and limits for safe consumption [3,4,5]. Worldwide, and in Brazil, caffeine intake occurs primarily through coffee consumption [1]. The estimation of the Brazilian population’s average daily coffee intake is 163 mL [6], being the most consumed non-alcoholic drink in Brazil [7,8]. In addition, caffeine is also widely consumed in other foods and beverages such as cola, cocoa, chocolate, guarana, and in matte, black, and green teas [9]. Furthermore, a range of energy drinks and sports supplements also contain caffeine in their composition [5]. The differences in biological individuality and cultural factors can influence the habits of caffeine consumption [10,11]. Therefore, the ingestion of products that contain caffeine is not only associated with their sensorial characteristics and eating habits but also with caffeine effect expectations [12].

It is well established that placebo effects are associated with caffeine supplementation, likely due to an expectancy surrounding its effects. Double-blind studies have shown that participants receiving a placebo treatment perceived to be caffeine improved exercise performance to a similar extent when compared with caffeine ingestion [13,14]. Positive expectation associated with caffeine ingestion appeared to drive this effect since individuals correctly believing that they had ingested caffeine improved to a greater extent than the average effect of caffeine [13,15]. However, these results were not observed in physiological variables, such as heart rate and blood pressure [16], further reinforcing the notion that the expected effect of caffeine plays a subjective role in the belief around its consumption [16,17]. Regarding expectancy, factors such as motivation and belief can influence the ergogenic response of caffeine in adults [17]. Therefore, expectancies associated with caffeine use/outcome may play an important role in the development, maintenance, and reinforcement of its consumption patterns [12,18,19]. Studies have attempted to associate habitual caffeine consumption with changes in mood, appetite, sleep/alertness, exercise performance, and other factors [19,20,21]. Based on these observations, standardized questionnaires were constructed using psychometric techniques [22] to assess expectancy about caffeine consumption [19,20,21], or to evaluate the motives for caffeine consumption [12].

In this regard, Heinz et al. (2009) [18] proposed a questionnaire with 37 items to examine caffeine expectancy comprising four factors: ‘withdrawal symptoms’, ‘positive effects’, ‘acute negative effects’, and ‘mood effects’. Subsequently, Huntley and Juliano (2012) [20] proposed the Caffeine Expectancy Questionnaire (CaffEQ), a structured questionnaire based on a detailed review of the literature and a series of preliminary studies for construction of the items. The final version of the CaffEQ (originally in English, designed for the United States of America) includes 47 items, evaluated using a six-point Likert scale, distributed across seven factors: ‘withdrawal/dependence’, ‘energy/work enhancement’, ‘appetite suppression’, ‘social/mood enhancement’, ‘physical performance enhancement’, ‘anxiety/negative physical effects’ and ‘sleep disturbance’. Besides its use in the English language, the CaffEQ was also translated and validated for German-speaking countries (Germany, Switzerland, and Austria) by the authors Schott et al. (2016) [21].

However, since the validation and standardization of the CaffEQ questionnaire were performed only for English and German speaking populations [19,20,21], there are currently no studies with Latin American countries using the CaffEQ due to linguistic barriers and cultural differences that cause difficulties in using the original questionnaire. In this sense, there has been no study proposed to evaluate caffeine expectations in the Brazilian population due to the lack of a valid questionnaire in the Brazilian-Portuguese language. Therefore, this study aimed to translate, culturally adapt, and validate the CaffEQ to the Brazilian population (CaffEQ-BR), and also to evaluate caffeine expectations in Brazilian adult participants. We expect that this study can provide a questionnaire with internal and external validity to characterize caffeine expectations in the Brazilian adult population and be an easy questionnaire to incorporate into research and clinical contexts.

## 2. Materials and Methods

The present study used the original CaffEQ and translated it from the English version to Brazilian-Portuguese [20]. The CaffEQ is composed of 47 items, evaluated using a six-point Likert scale. In order to create the CaffEQ for the Brazilian population (CaffEQ-BR), our study was conducted in four stages: (1) Translation, Cultural Adaptation, and Semantic Evaluation; (2) Internal Consistency and Reproducibility of CaffEQ-BR; (3) Brazilian nationwide CaffEQ-BR application; (4) Statistical analysis. The study was approved by the Ethics Committee of the University Católica of Brasília (Brasília, Brazil) (number: 23019319.3.0000.0029) and followed the guidelines established by the Declaration of Helsinki. The volunteers were informed about the study protocol and provided web-based consent.

In the present study, the survey was carried out using Google Forms™ web-based platform [23]. The online form maintained the original CaffEQ version layout and content [20]. The expert panel suggested inserting an explanation about the meaning of the word caffeine (as well as about its main sources) in the questionnaire heading for a better understanding of the questionnaire by the general public, since the term “caffeine” is not common to the Brazilian population.

### 2.1. Translation, Cultural Adaptation, and Semantic Assessment

The translation and cultural adaptation of the questionnaire was performed according to World Health Organization (WHO) recommendations [24]. A bilingual researcher native in Portuguese (T.H.M.d.C.) translated the original version (in English) of the CaffEQ into the Brazilian-Portuguese language. Subsequently, another bilingual researcher, a native English speaker (resident in Brazil for eight years) (B.S.), with no knowledge of the original work, back-translated the Brazilian-Portuguese version (made by T.H.M.d.C.) into English. After that, three collaborators (G.F.M.; C.E.G.R.; R.P.Z.) compared the back-translated version (in English, made by B.S.) with the original questionnaire and analyzed the Brazilian-Portuguese translation version to make adjustments in case of non-conformities. The final version was agreed upon by the bilingual translators (T.H.M.d.C. and B.S.) as a final step in the translation process.

The questionnaire was subsequently analyzed and revised by a panel of health professional experts (*n* = 20) distributed across the following academic degrees: Master’s (*n* = 7; 35%), Doctorate (*n* = 9; 45%) and Post-doctorate (*n* = 4; 20%), all associated with universities and all residents in Brasília Federal District [22]. The experts individually analyzed the cultural adaptation and semantic assessment using parameters of the ‘importance’ and ‘clarity’ of each question (*n* = 47) on a Likert scale of 1 to 5, where 1 indicates “I totally disagree with the item”; 2—“I partially disagree with the item”; 3—“I neither agree nor disagree with the item”; 4—“I partially agree with the item”; and 5—“I fully agree with the item”. The objective was to achieve more than 80% agreement among the experts (mean > 3) for each question [25,26]. Pending items were adjusted according to the experts’ observations and sent back to them for compliance analysis. This process occurred until all items achieved at least 80% agreement (mean > 3). The degree of agreement among experts in the evaluation of the ‘importance’ and ‘clarity’ of the questions was performed by the Kendall correlation coefficient (W) ranging from 0 to 1. A W-value ≥ 0.66 indicates that the experts applied the same evaluation standards, and W-values < 0.66 suggest disagreement between experts. To approve an item, it was deemed necessary that at least 80% agreement was achieved among the experts (W values ≥ 0.8) [26].

### 2.2. Internal Consistency and Reproducibility of CaffEQ-BR

The reproducibility of the translated and adapted instrument CaffEQ-BR was analyzed before nationwide application since, before application in a large sample, it is important to test the reproducibility (reliability) and internal consistency with a small sample size [27]. Internal consistency refers to the variation in measurements made under changing conditions and reproducibility evaluates the agreement between any two measurements made on the same subject [27].

For this purpose, the questionnaire was applied using the Google Forms™ platform to a convenience sample (*n* = 50) of Brazilian adults (>19–59 y) who were regular consumers of caffeine from various sources. Participants were invited through pilot advertising on social media (for example, Facebook™, Instagram™, and WhatsApp™). The questionnaire was answered twice (test-retest) by each person. The second questionnaire was sent within 24 h and returned within the next 24 h. The test-retest questionnaires evaluated reproducibility. It is important to note that the participants did not previously know that they would have to answer the questionnaire a second time. The test-retest reliability (reproducibility) analysis was performed using the intraclass correlation coefficient (ICC), and the internal consistency of the factors was verified using Cronbach’s alpha (α). The number of individuals used in this step was considered sufficient once the results were statistically significant (*p* < 0.05) and the effect size was significant (alpha > 0.9 and ICC > 0.6) [28,29].

### 2.3. Brazilian Nationwide Application of CaffEQ-BR

In order to validate the CaffEQ-BR in Brazil and also to evaluate the Brazilian adult population, we used a questionnaire composed of three parts: (i) sociodemographic and health-related questions; (ii) evaluation of caffeine consumption; and (iii) the CaffEQ-BR. According to Hair et al. (2010) [30], the process of validating a questionnaire requires 20 respondents per item (20:1). In this sense, the minimum sample size was estimated as 940 participants to validate this questionnaire composed of 47 items. In addition, as this is a nationally external validation study, the sample size adopted for calculation was in accordance with the last Brazilian national census [26], with adequacy greater than or equal to 70% of the sample distribution, according to the various states of Brazil. In the example of the state of Rio de Janeiro, the population of 17,264,943, represents 8.22% of the population of Brazil. Therefore, the CaffEQ-BR sample, to obtain 100% adequacy, must have 8.22% of its total sample composed of participants from the state of Rio de Janeiro. In this way, we balanced the sample among the states of Brazil.

The questionnaire was applied using the Google Forms™ platform to a convenience sample of Brazilian adults from all 27 Brazilian states. Participants were recruited by advertising on social media (e.g., Facebook™, Instagram™, and WhatsApp™) [21]. The data collection period occurred from December 2019 to April 2020.

The initial page of the online survey presented the informed consent form with details of the inclusion criteria: (i) adults (>19–59 y) [31,32]) living in Brazil; (ii) regular consumer of caffeine sources (at least three times per week [33]), later confirmed by the caffeine consumption questionnaire. Those who did not agree to participate were directed to a page thanking them for their time, while those who agreed were directed to the first page of the questionnaire with sociodemographic and health-related questions, then caffeine consumption assessment and the 47-item CaffEQ-BR.

#### 2.3.1. Sociodemographic and Health Data

Sociodemographic variables were gender; self-identification of ethnicity; state of the federation of current residence; education level; and average monthly income (BRL/month/person or family). The variables concerning health aspects were height (m) and weight (kg) (self-reported); ≥150 min weekly physical exercise; and previous diagnosis of self-reported chronic diseases with current medication.

#### 2.3.2. Caffeine Consumption 

The caffeine consumption questionnaire [33,34] was used to assess the caffeine consumed over the past two weeks prior to the completion of the questionnaire. Participants were asked to indicate the number of servings of coffee, tea, soft drinks, energy drinks, and other caffeine-containing products consumed. The questionnaire also includes a list categorized into eight groups of caffeine sources: 1. Filtered or espresso, hot or iced coffee; 2. Tea sources of caffeine like mate, green and black tea; 3. Pure chocolate with 50% cocoa; 4. Chocolate beverages with 50% cocoa; 5. Cola or guarana based soft drinks; 6. Caffeinated drugs; 7. Commercial drink sources of anhydrous caffeine or guarana extract beverage; 8. Sports supplements sources of anhydrous caffeine. Standardized doses of coffee, in homemade measures, were adopted according to the national reference study [35]. The typical serving size and caffeine values were based on the products’ manufacturer information and the food composition table [36].

### 2.4. Statistical Analysis

A confirmatory factor analysis verified the factor validity. The factor validity was evaluated by the Root Mean Square Error of Approximation (RMSEA). The RMSEA ranges from 0 to 1, where the value 0 indicates a perfect model fit. A value of 0.05 or less is indicative of an acceptable model fit. Caffeine intake was expressed as a mean ± standard deviation (SD). Shapiro-Wilk test was used to evaluate the normality of distribution. The independent samples t-test was used to compare means between gender. All tests were conducted considering a significance level of 5%. The statistical packages IBM SPSS (Statistical Package for Social Sciences) version 22 (IBM SPSS Statistics for Windows, IBM Corp, Armonk, NY, USA) and IBM SPSS AMOS (Analysis of Moment Structures) version 22 (Amos, IBM SPSS, Chicago, IL, USA) were used for the analyses.

## 3. Results

### 3.1. Translation, Cultural Adaptation, Semantic Evaluation, and Content Validation

**Figure 1:**
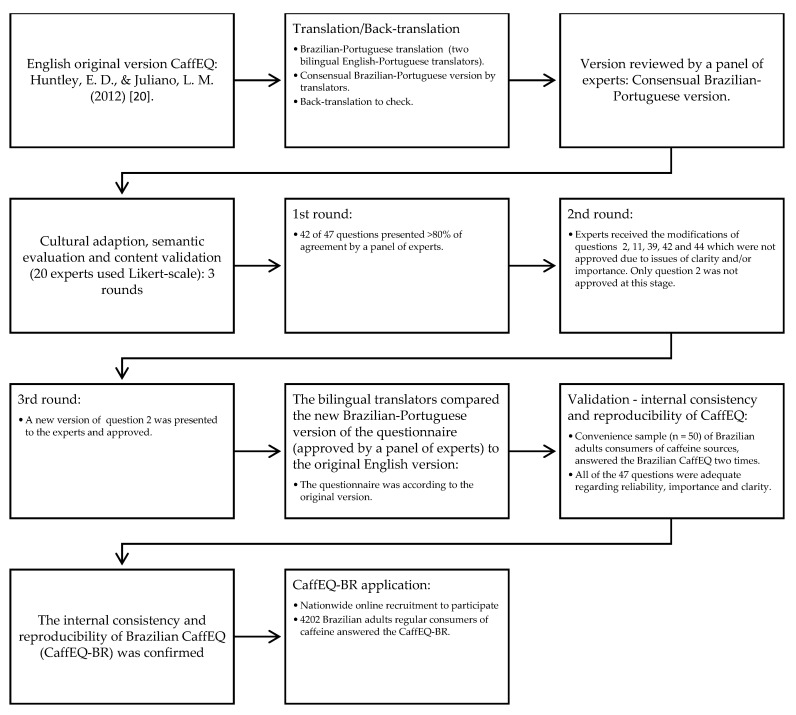
Flowchart of translation, cultural adaptation, semantic evaluation, content validation processes and application of the Caffeine Expectancy Questionnaire in Brazil (CaffEQ-BR).

The CaffEQ-BR (available in Brazilian-Portuguese in Appendix A) was constructed considering the translation/back-translation process and the suggestions made by the expert panel. Following the translation/back-translation phase, the first stage of semantic evaluation and content validation was carried out by the panel of 20 experts who decided to keep 47 items with cultural and semantic adaptations, since we chose to follow the original CaffEQ questionnaire [20]. Throughout three rounds of assessment, with modifications in the items regarding cultural and semantic aspects, the experts reached agreement (≥80%) on the evaluation of the 47 items in the questionnaire. After that, with a convenience sample of 50 Brazilian adults (60% female, 36.4 ± 12.4 y, 62.2% self-identification as white), the internal consistency and reproducibility of CaffEQ-BR were verified. A summary of the translation, cultural adaptation, semantic evaluation, and content validation processes for CaffEQ-BR is shown in Figure 1.

### 3.2. Reproducibility and Internal Consistency of the CaffEQ-BR

All seven factors of the CaffEQ-BR showed no significant difference (ICC > 0.9) in the responses from the same individual (*n* = 50) (Table 1). As shown in Table 1, all seven factors indicated good internal consistency (α ≥ 0.8) [29,37].

### 3.3. Brazilian Nationwide Application of the CaffEQ-BR

#### 3.3.1. Participants

**Figure 2:**
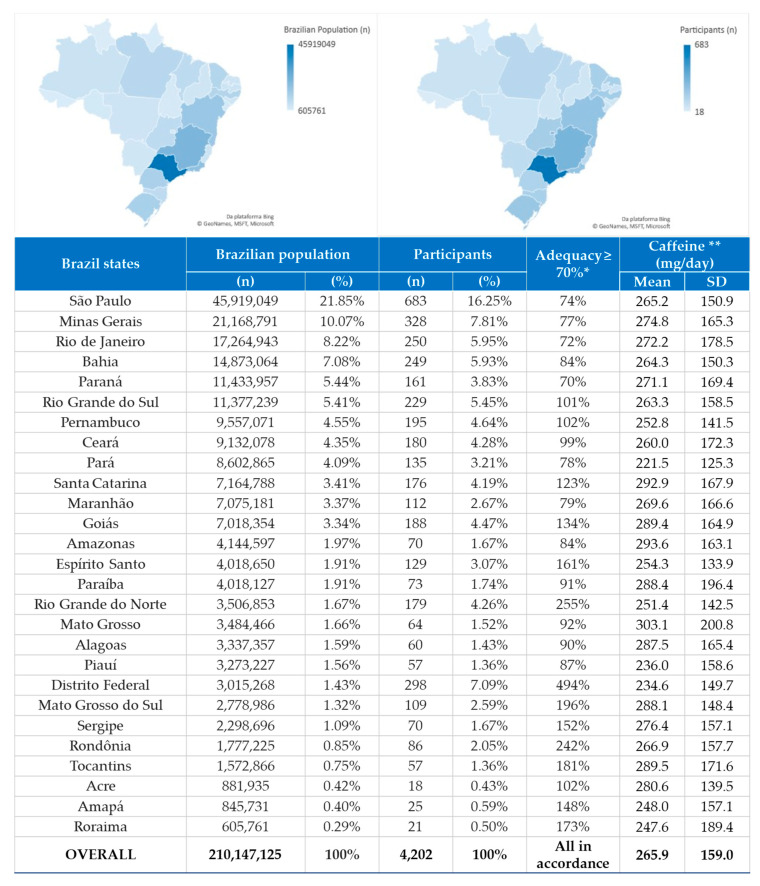
National distribution of participants and average caffeine consumption. * As this is an external validation study of national scope, the sample calculation was performed according to the last Brazilian national census [38], with adequacy greater than or equal to 70% of the sample distribution according to the states of Brazil; ** caffeine in general sources. Northeast Region-Alagoas, Bahia, Ceará, Maranhão, Paraíba, Pernambuco, Piauí, Rio Grande do Norte, Sergipe; North Region-Acre, Amazonas, Amapá, Pará, Rondônia, Roraima, Tocantins; Midwest Region-Distrito Federal, Goiás, Mato Grosso, Mato Grosso do Sul; South Region-Paraná, Santa Catarina, Rio Grande do Sul; Southeast Region-Espírito Santo, Minas Gerais, Rio de Janeiro, São Paulo.

From 4339 individuals who responded to the online CaffEQ-BR questionnaire, the final sample was composed of 4202 participants, since some participants (*n* = 137) did not provide all the data necessary for their inclusion in the survey. The nationwide distribution of the participants among the Brazilian states is presented in Figure 2. Participants were mostly from the Southeast Brazilian region (*n* = 1390; 33.08%), followed by the Northeast (*n* = 1175; 27.96%), Midwest (*n* = 716; 17.04%), South (*n* = 566; 13.47%) and North (*n* = 355; 8.45%). The state with the highest participation was São Paulo-Southeast region (*n* = 683; 16.25%), and the lowest was Acre-North region (*n* = 18; 0.43%). Figure 2 shows the methodological rigor of adequacy of 70% or more in the sample representation, according to the last national census [38], since all Brazilian states achieved this goal. Figure 2 also displays the mean of participants’ caffeine and coffee consumption by each Brazilian state.

Table 2 shows the balanced distribution of participants by gender, with the highest frequency of participants aged between 31 and 59 y (*n* = 2625; 62.5%) and the majority of people of normal weight (classified by BMI between 18.5–24.9 kg/m^2^ [31]). More than half of the sample (*n* = 2328; 55.4%) was white, as well as physically active (*n* = 2278; 54.2%). Graduates and postgraduates were the most frequent educational level (*n* = 2477; 59%). A monthly income between 3000.01 and 5000.00 (BRL) was the most frequent (*n* = 865; 20.6%). A large part of the sample did not report having any chronic disease (*n* = 3397; 80.8%). More information on sociodemographic aspects is shown in Table 2.

#### 3.3.2. Caffeine Consumption

Based on weekly consumption of caffeine sources, the average daily intake observed was 265 ± 159 mg (minimum 49 mg; maximum 1200 mg). The total caffeine intake for males (274 ± 162 mg/day) and for females (256 ± 155 mg/day) was statistically different (t = 3703; df = 4200; *p* < 0.001). Figure 2 shows descriptive data of average caffeine consumption by Brazilian states. A very similar pattern of consumption was observed between states. The average consumption by regions was as follows: North (*n* = 355; caffeine consumption: 253 ± 150 mg/day); Northeast (*n* = 1175; caffeine consumption: 262 ± 157 mg/day); Midwest (*n* = 716; caffeine consumption: 267 ± 162 mg/day); Southeast (*n* = 1390; caffeine consumption: 267 ± 158 mg/day); South (*n* = 566; caffeine consumption: 274 ± 164 mg/day). Thus, the highest absolute consumption of caffeine was in the southern region. Table 3 shows the distribution of consumption of caffeine sources and the time of the day that these were consumed. 

The participants’ main source of caffeine was coffee, mostly consumed in the morning. In the afternoon, soft drinks and chocolates were the primary sources of caffeine. In the evening, the consumption of coffee, teas, chocolate, soft drinks, caffeine medications, and energy drinks was more frequent. Chocolate beverages showed no difference in consumption during the day. Caffeine-based sports supplements were most frequent in the morning. Coffee was the only source of caffeine with a predominance (73.3%) of consumption of two or more servings daily.

#### 3.3.3. Confirmatory Factor Analysis and Associations of the CaffEQ-BR

Based on the national sample (*n* = 4202), the external factor validity of CaffEQ-BR was verified by confirmatory factor analysis. The seven factors presented RMSEA = 0.0332 (95% CI: 0.0290–0.0375), which shows satisfactory external validity.

Table 4 shows the results of the Pearson correlation coefficient between the CaffEQ-BR scores, divided between the seven factors, and the consumption of caffeine. All correlation between caffeine consumption and CaffEQ-BR factors (F1 to F6) were positive weak (*r* < 0.4) and significant (*p* < 0.001), except for F7 (−0.074; *p* < 0.001). Therefore, the higher the consumption, the higher the score. Despite the weak correlation (*r* < 0.4), the association between caffeine consumption and the CaffEQ-BR scores were all significant (*p* < 0.001), due to the large sample size (*n* = 4202).

In Table 5, the region of Brazil with the highest average for F1 factor (withdrawal/dependence) was the southeast. The F2 (energy/work) enhancement factor resulted in the highest average score among all factors and was similar between regions in Brazil. The F3 factor (appetite suppression) was below 3 on the six-point Likert scale for all regions of Brazil. The factors F4 (social/mood enhancement) and F5 (physical performance enhancement) were above 3 on the Likert scale, with emphasis on the upper average for F4 in the north region, and the lower average for F5 in the south region of Brazil. The F6 factor (anxiety/negative physical effects) resulted in the lowest average score among all factors, with a similarity between regions. The F7 factor (sleep disturbance) was also below 3 on the Likert scale, with the lowest average for the south region of Brazil.

## 4. Discussion

In this original study, we developed and validated the Brazilian version of the CaffEQ. Until now, there has been no adaptation of CaffEQ to Brazilian-Portuguese in the cultural context of Brazil, or in Latin American countries. Its application may assist in observational studies for clinical trials that assess caffeine consumption in Brazil. The selected questionnaire also allowed us to make comparisons with data available from other countries that used the same questionnaire [19,20,21]. The CaffEQ-BR is a questionnaire designed to identify the expectations that Brazilian individuals have about the subjective effects of caffeine on the biopsychosocial aspects involved in its consumption [19,20,21].

In order to create the CaffEQ-BR, the translation and back-translation process (linguistic validation of the instrument) was necessary, since the original questionnaire was developed in another language and there was no translated and validated version in the target language [24]. Therefore, the first step of this study was to translate/retranslate the original version of CaffEQ from English to Brazilian-Portuguese to English following the scientific guidelines proposed by the WHO [12,24]. After this, the questionnaire was sent to experts for evaluation, since semantic evaluation is necessary to ensure its clarity and comprehension [40,41]. In this sense, CaffEQ-BR presented cultural and semantic adequacy according to the consensus of the experts (at least 80% of agreement). After this stage, the test-retest with 50 individuals was used to assess the reliability of the CaffEQ-BR, which analyzes the questionnaire’s ability to reproduce consistent results [29,41]. The internal consistency of CaffEQ-BR was measured by Cronbach’s alpha coefficient (α = 0.94), considered acceptable when ≥0.8 [28,37]. This result was similar to the findings of Huntley and Juliano (2012) [20] (*n* = 1046; α = 0.96), and Schott et al. [21] (*n* = 352; α = 0.98) for the same questionnaire in English and German, respectively. In addition, the CaffEQ-BR presented excellent measures of reproducibility (ICC = 0.97). This result confirms that the questionnaire is able to consistently measure the subjective effects of caffeine perceived by the interviewed user. Every scale used to measure health results needs this reliability performed by exploratory and confirmatory factor analysis [37].

After internal validation of the CaffEQ-BR, we conducted a national study in Brazil, using a sample of all 27 Brazilian states (Figure 2) with uniform distribution of age and sex (Table 2), similar to the last available national census (Brazil-IBGE (Instituto Brasileiro de Geografia e Estatística) 2010) [42]. The national census is usually held every decade, and the 2020 edition is in progress. The first five most populous Brazilian states (Figure 2) had two or more rounds of dissemination of the survey on social networks, to achieve the established interview number goal. The Federal District had the highest representation in percentage points because the research group is based in Brasília, Federal District. Naturally, in a convenience sample, there was greater participation in our hometown.

The sociodemographic data of the CaffEQ-BR participants are closer to the measures of the adult Brazilian population on gender and age than the sample of the original study (CaffEQ) [20], which had a predominance of young female students. Although the CaffEQ-BR sample is representative of the population distribution parameters in the Brazilian states [38], there is a selection bias in relation to the respondents’ education and socioeconomic level, which was above the national average family income (1439 BRL per month in 2019) [43] directly influencing educational status [44]. Another factor was the use of social networks to disseminate the research questionnaire. Other nationwide surveys in Brazil from our institution/research groups, released through web-base, also observed greater access by higher economic classes compared to the national average [45,46]. Therefore, it is not possible to extrapolate our results to the entire Brazilian adult population. This is not a national census or national sample representation.

Regarding the self-reported categories for BMI ≥ 25 and chronic disease being treated, our sample data showed a lower incidence of these two variables (55.8%; 19.2%, respectively) compared to the results of the Brazilian national study “Surveillance of risk and protective factors for chronic diseases by telephone research” (VIGITEL 2019) (75.7%; 31.9%, respectively). However, our sample showed a higher frequency of people who self-reported being physically active: 54.2%, compared to the last VIGITEL (2019) which showed 39.5% [47]. The VIGITEL study used a representative random sample only from the state capitals of Brazil, through phone interviews. Our survey did not cover capitals only, with a convenience sample by invitation on social networks with predominant access via mobiles. There was an inclination towards greater sample composition of middle-aged adults, with a higher level of education and income for the CaffEQ-BR. There are studies that indicate a greater preference, especially for coffee, in individuals with this sociodemographic profile [6,7,8]. The VIGITEL study in different periods of time (2006 to 2019) showed that that part of the population that has more years of schooling (≥12 years) is less overweight, sedentary, and chronically ill. The reverse context, low income and education level and high morbidity rate, are also observed [48]. The self-reported ethnicity comparison between the National Household Sample Survey (2018/2019) [43] and that obtained in the CaffEQ-BR was: 45.2–55.4% of Brazilians that declared themselves as white, 45.0–31.6% as pardo, 8.8–8% as black, 0.47–2.7% as Asian descendants and 0.38–1.0% as indigenous.

The average caffeine intake in our sample (265 ± 159 mg/day) is above the published standards for Brazil (115 ± 96 mg/day) [49]. According to Sartori et al., the survey on caffeine consumption in Brazil was based on food sources, extracted from data from the national survey of 2008 and 2009 [49]. In addition to the difference in the observation periods (2008/09 vs. 2019/20), our survey included other sources of caffeine as supplements and medications. This fact is relevant according to Arrais et al. (2016) [50], as self-medication is a recurrent practice in Brazil, including among young adults, mainly associated with the use of non-prescription medications, such as analgesics and muscle relaxants. In the national market, these drugs take in their composition, on average, 30 to 50 mg of caffeine per serving. Our study also focused on individuals who are regular consumers of caffeine (from different sources); therefore, we expect that the participants’ average usual intake could be higher than the general Brazilian population. These values were similar to those found by Schott et al. (from Germany, Switzerland, and Austria: 236 ± 235 mg/day) [21] but considerably below the consumption found by Huntley and Juliano (from U.S.: 323 ± 297 mg/day), which was based on the consumption of a younger population, containing many college/university students [20]. Another point is that the volume of coffee consumed in Brazil is not a standard variable to be compared with a North American or European study, since Brazilians and inhabitants of other Latin American countries usually drink small portions of stronger coffee (approx. 50 mL of small cups) compared to the American culture of large cups (approx. 250 mL) of lighter coffee, a fact observed by De Paula and Farah (2019) [51]. Total caffeine intake in males (274 ± 162 mg/day) was higher than in females (256 ± 155 mg/day), similar to the results observed by other studies [8,49,52]. Probably these gender differences are related to cultural and behavioral factors in males as well as to the gender differences in physiological responses to caffeine [53,54,55,56]. A study showed that males differ in cardiovascular responses to caffeine, while females did not differ in their responses as a function of typical caffeine use [55]. Males also presented greater decreases in heart rate in response to caffeine than did females, probably related to changes in circulating steroid hormone, in which increased circulating estradiol increases the physiological and subjective effects associated with caffeine, influencing the high consumption of caffeine on males [53].

Across previous CaffEQ studies, caffeine was consumed mainly in coffee, a habit also observed in Brazil [6,7,8]. Globally, habitual coffee consumption ranges from about 1 to more than 5 cups per day, which indicates that the daily dose is defined by several reasons, like lifestyle, gender, expectance of caffeine effects, culture, genetics, health effects, among others [2,36,57]. Most of our sample (69%) are used to consuming 2 or 3 portions of coffee daily (Table 3). The culture of coffee in Brazil has a historical origin in its production capacity, as it is the largest coffee exporter in the world market [58]. Brazil accounts for one-third of the world’s coffee production, making it the world’s largest producer, a position it has occupied for more than 150 years. In Brazil, at the beginning of the 19th century, coffee was already treated as an investment. With the expansion of plantations in the country, there was also an expansion of investment favoring urbanization, such as the construction of railroads responsible for the national distribution and export of coffee, in addition to the arrival of immigrants. Thus, in Brazil, coffee is considered one of those products responsible for the modernization, urbanization and development of some cities [59], and it is still widely consumed and appreciated throughout the country. Annual per capita Brazilian consumption is 6.02 kg, which represents 13% of world demand [60]. Easy access to coffee naturally influences the consumption culture of Brazilians [8]. Coffee is the main drink consumed, with an average of 163 mL per day, and is also the second most consumed food [6,7].

In Brazil, coffee consumption is widespread [6]. This reflects a very similar average consumption between regions [6], as observed in the CaffEQ-BR survey. The differences are greater when other eating habits are associated with the daily use of coffee. For example, the habit of consuming a hot mate called “Chimarrão” in the south region, a cold mate called “Terere” in the Midwest region [61] and guarana extract in the northern region of Brazil [62]. The fact that the Northeast region is the largest consumer of coffee was also confirmed by the study of Sousa and Da Costa [6]. We also emphasize that coffee and other caffeine sources are also sources of other bioactive compounds, including polyphenols and chlorogenic acids [63]. However, the main substance with psychoactive properties is caffeine, confirmed by several meta-analyses [4]. The construction of the original CaffEQ [20] takes into account the estimated average consumption of caffeine in general (from all sources), without the intention of associating it with other compounds present in food sources of caffeine.

The statistical correlations (*r* < 0.4) shown between CaffEQ factors, scores and caffeine consumption were also observed in previous studies that used the original CaffEQ in the United States [20] and the translated and validated version in German-speaking countries [21].

When observing the descriptive results of the CaffEQ-BR scores divided into seven factors using the original questionnaire [20], it is possible to observe similarity in the factors Withdrawal/dependence 3.48 (1.43)–3.22 (1.45), Energy/work enhancement 4.14 (1.32)–3.92 (1.17), Appetite suppression 2.24 (1.17)–2.70 (1.20), Social/mood enhancement 3.41 (1.38)–2.98 (1.21), respectively. However, there was a difference of approximately one point for the factors Physical performance enhancement 3.47 (1.51)–2.41 (1.07), Anxiety/negative physical effects 1.78 (0.77)–2.68 (1.04) and Sleep disturbance 2.47 (1.62)–3.20 (1.45). Differences in mean scores in the seven factors were also observed in the other cultures where CaffEQ was studied [20,21].

Data from the latest survey published by the Brazilian Institute of Geography and Statistics showed that three out of four Brazilians in metropolitan capitals (Belém (Pará), Fortaleza (Ceará), Recife (Pernambuco), Salvador (Bahia), Belo Horizonte (Minas Gerais), Rio (Rio de Janeiro), São Paulo (São Paulo), Curitiba (Paraná) and Porto Alegre (Rio Grande do Sul)) have access to the Internet, and the number of households with landlines dropped from 33.6 % to 31.5%, while ownership of devices with mobile internet increased from 92.6% to 93.2% [64]. The smartphone was also the main tool used to access the internet. Therefore, although web-based research may be limited because it is not possible to reach every portion of the population, it can still be considered a viable strategy since our web search could be answered on any device with internet. There is also the limitation of memory and intake bias, which is intrinsically related to frequency questionnaires [65].

There is no other scientifically validated Brazilian research questionnaire that evaluates consumption related to caffeine. Therefore, there are no parameters for comparison except with the original version of the CaffEQ [20] and the German version [21]. Another important factor is the heterogeneity of the Brazilian-Portuguese language in the national territory. Certainly, there are aspects of regionality, but despite these limitations, due to the construction process in several stages and the wide statistical confirmation, the Brazilian version of CaffEQ represents a reliable and valid questionnaire to assess expectations of caffeine intake. Analytical item analysis confirms the quality of the translated items. Overall, the CaffEQ’s translation and validation for Portuguese and Brazilian culture were successful.

## 5. Conclusions

The full version of the Caffeine Expectancy Questionnaire in Brazil (CaffEQ-BR) is available for Brazilian adults, translated into Portuguese and adapted to Brazilian culture. This study confirmed the validity and reliability of the CaffEQ-BR. Its internal and external consistency allows its use throughout the national territory, if the sampling conditions are similar. The CaffEQ-BR observed the pattern of consumption of caffeine sources by Brazilian adults, confirming the national preference for coffee as the main source of daily caffeine. Future studies may validate the CaffEQ-BR in children, adolescents and the elderly, since caffeine is widely consumed across the lifespan. The present study contributes to a better understanding of the expectations of the most used psychoactive substance in Brazil, systematizing several expectations in seven factors that can be explored and categorized. Thus, the CaffEQ-BR can be used to facilitate our understanding of the use of caffeine. Other studies may also replicate our results, pointing out the temporal stability of the CaffEQ-BR, monitoring changes in expectations in longitudinal exposure to caffeine.

## Figures and Tables

**Table 1:** Reproducibility and internal consistency of the instrument and factors of the Caffeine Expectancy Questionnaire in Brazil (CaffEQ-BR) *.

Factors	N. Items	Internal Consistency Cronbach Alpha (95% CI)	Reproducibility Intraclass Correlation Coefficient (95% CI)
Withdrawal/dependence	12	0.948 (0.923–0.968)	0.983 (0.969–0.991)
Energy/work enhancement	8	0.926 (0.888–0.923)	0.953 (0.912–0.975)
Appetite suppression	5	0.872 (0.802–0.923)	0.951 (0.903–0.974)
Social/mood enhancement	6	0.889 (0.829–0.932)	0.949 (0.900–0.973)
Physical performance enhancement	3	0.924 (0.875–0.956)	0.965 (0.936–0.981)
Anxiety/negative physical effects	9	0.872 (0.807–0.921)	0.953 (0.907–0.976)
Sleep disturbance	4	0.941 (0.907–0.965)	0.970 (0.945–0.983)
**Overall**	**47**	**0.948 (0.923–0.967)**	**0.976 (0.935–0.989)**

* For reproducibility and internal consistency of items and factors of the CaffEQ-BR, conducted with a convenience sample of 50 Brazilian adults: 60% female, 36.4 ± 12.4 y, 62.2% of self- identification as white.

**Table 2:** Sociodemographic data, sample profile of the CaffEQ-BR study (2019–2020).

	Categories	Total (*n* = 4202)	
		*n*	%
**Gender**	Male	2063	49.1
	Female	2139	50.9
**Age**	19–24	822	19.5
	25–30	755	18.0
	31–40	1331	31.7
	41–59	1294	30.8
Body Mass Index * (kg/m²)	<18.5	106	2.5
	18.5–24.9	1751	41.7
	25–29.9	1498	35.6
	≥30	847	20.2
**Self-Identified ethnicity**	Asia descendants	114	2.7
	White	2328	55.4
	Indigenous	41	1.0
	Pardo	1330	31.6
	Black	309	7.4
	Without description	80	1.9
**Physical Exercises ≥ 150 min/week**	No	1924	45.8
	Yes	2278	54.2
**Educational Level**	No schooling	3	0.1
	Incomplete elementary school	17	0.4
	Completed elementary school	37	0.9
	Incomplete high school	101	2.4
	Completed high school	596	14.2
	Incomplete higher education	955	22.7
	Higher education graduate	1162	27.6
	Postgraduate studies	1315	31.3
	Without description	16	0.4
Monthly Income (BRL) **	1000.00	407	9.7
	1000.01 to 2000.00	769	18.3
	2000.01 to 3000.00	669	15.9
	3000.01 to 5000.00	865	20.6
	5000.01 to 10,000.00	796	18.9
	Above 10,000.00	575	13.7
	Without description	121	2.9
**Self-Reported Chronic Diseases**	No	3397	80.8
	Yes	805	19.2

* Body mass index (BMI) followed the criteria adopted by the World Health Organization (WHO) [39] underweight (BMI < 18.5 kg/m^2^), adequate (BMI between 18.5 and 24.9 kg/m^2^), overweight (BMI between 25 and 29.9 kg/m^2^) and obesity (BMI ≥ 30 kg/m^2^). ** 5.55 BRL = 1.00 USD on the last day of data collection, April 2020.

**Table 3:** Distribution frequency of regular consumption of sources of caffeine per week (*n* = 4202).

Caffeine Sources ¹		Coffee ^2^		Tea ^3^		Chocolate ^4^		Chocolate Beverages ^5^		Soft Drinks ^6^		Medication ^7^		Energy Drinks ^8^		Sports Supplements ^9^	
		N	(%)	N	(%)	N	(%)	N	(%)	N	(%)	N	(%)	N	(%)	N	(%)
Time of the Day	Early morning (00:00–06:00)	202	4.8%	79	1.9%	153	3.6%	70	1.7%	149	3.5%	115	2.7%	109	2.6%	19	0.5%
	Morning (06:00–12:00)	3853	91.7%	503	12.0%	644	15.3%	653	15.5%	362	8.6%	505	12.0%	188	4.5%	333	7.9%
	Afternoon (12:00–18:00)	2829	67.3%	686	16.3%	1594	37.9%	523	12.4%	1361	32.4%	396	9.4%	324	7.7%	206	4.9%
	Evening (18:00–24:00)	1508	35.9%	878	20.9%	1291	30.7%	563	13.4%	1058	25.2%	699	16.6%	348	8.3%	122	2.9%
N° of Servings Per Day	1	971	23.1%	1178	28.0%	1647	39.2%	908	21.6%	985	23.4%	824	19.6%	530	12.6%	463	11.0%
	2	1958	46.6%	322	7.7%	537	12.8%	273	6.5%	543	12.9%	209	5.0%	141	3.4%	78	1.9%
	3	983	23.4%	92	2.2%	235	5.6%	89	2.1%	209	5.0%	111	2.6%	43	1.0%	19	0.5%
	4	139	3.3%	12	0.3%	64	1.5%	22	0.5%	58	1.4%	35	0.8%	7	0.2%	1	0.0%
Total Recorded		4051	96.4%	1604	38.2%	2483	59.1%	1292	30.7%	1795	42.7%	1179	28.1%	721	17.2%	561	13.4%

^1^ Standardization of the portions in the consumption frequency table was adopted based on the dose of 50 mg of caffeine/portion. The percentages exceed 100% because consumption can occur in two or more periods. ^2^ Filtered or espresso, hot or iced coffee. ^3^ Tea sources of caffeine like mate, green and black tea. ^4^ Pure chocolate with ≥ 50% cocoa. ^5^ Chocolate beverages with ≥ 50% cocoa. ^6^ Cola nut or guarana based soft drinks. ^7^ Caffeinated medications. ^8^ Commercial drink sources of anhydrous caffeine or guarana extract beverage. ^9^ Sports supplements sources of anhydrous caffeine.

**Table 4:** Correlations Between Caffeine Expectancy Questionnaire in Brazil (CaffEQ-BR) Factors and Caffeine-Related Variables (*n* = 4202).

Sources	Factors of the CaffEQ-BR *						
	F1	F2	F3	F4	F5	F6	F7
Caffeine ** (mg/day)	0.085 ***	0.102 ***	0.081 ***	0.141 ***	0.097 ***	0.095 ***	−0.074 ***

* Factors of the CaffEQ-BR: F1 Withdrawal/dependence; F2 Energy/work enhancement; F3 Appetite suppression; F4 Social/mood enhancement; F5 Physical performance enhancement; F6 Anxiety/negative physical effects; F7 Sleep disturbance; ** Caffeine in general sources (Tea, coffee, chocolate above 50% cocoa, chocolate beverages, cola nut or guarana based soft drinks, caffeinated drugs, commercial drinks and sports supplements sources of anhydrous caffeine or guarana extract beverage); Pearson correlation *** *p* < 0.001.

**Table 5:** Mean and Standard Deviation (SD) of the scores on a six-point Likert scale of the seven factors of the Caffeine Expectancy Questionnaire in Brazil (CaffEQ-BR) by regions of Brazil (*n* = 4202).

Regions **	Factors of the CaffEQ-BR * Mean (SD)						
	F1	F2	F3	F4	F5	F6	F7
North	3.48 (1.49)	4.16 (1.37)	2.21 (1.15)	3.56 (1.45)	3.49 (1.55)	1.78 (0.69)	2.51 (1.60)
Northeast	3.44 (1.41)	4.15 (1.31)	2.24 (1.14)	3.44 (1.38)	3.55 (1.53)	1.81 (0.77)	2.45 (1.58)
Midwest	3.34 (1.39)	4.08 (1.32)	2.13 (1.14)	3.25 (1.34)	3.50 (1.53)	1.85 (0.82)	2.62 (1.69)
Southeast	3.60 (1.45)	4.17 (1.33)	2.26 (1.18)	3.41 (1.38)	3.47 (1.49)	1.75 (0.75)	2.44 (1.62)
South	3.47 (1.43)	4.08 (1.30)	2.36 (1.24)	3.42 (1.34)	3.24 (1.48)	1.74 (0.75)	2.36 (1.57)
Brazil	3.48 (1.43)	4.14 (1.32)	2.24 (1.17)	3.41 (1.38)	3.47 (1.51)	1.78 (0.77)	2.47 (1.62)

* Factors of the CaffEQ-BR, range: 1.00–6.00: F1 Withdrawal/dependence; F2 Energy/work enhancement; F3 Appetite suppression; F4 Social/mood enhancement; F5 Physical performance enhancement; F6 Anxiety/negative physical effects; F7 Sleep disturbance. ** Regions of Brazil: North Region-Acre, Amazonas, Amapá, Pará, Rondônia, Roraima, Tocantins; Northeast Region-Alagoas, Bahia, Ceará, Maranhão, Paraíba, Pernambuco, Piauí, Rio Grande do Norte, Sergipe; Midwest Region-Distrito Federal, Goiás, Mato Grosso, Mato Grosso do Sul; South Region-Paraná, Santa Catarina, Rio Grande do Sul; Southeast Region-Espírito Santo, Minas Gerais, Rio de Janeiro, São Paulo.

## Appendix A

**Questionário de Expectativa de efeitos da Cafeína/café,** **versão brasileira (CaffEQ-BR)**

Instruções: Estamos interessados em suas crenças sobre os efeitos que a cafeína tem sobre você. Abaixo há uma lista de possíveis efeitos da cafeína presentes nos produtos listados na tabela acima preenchida. Usando a escala como guia, avalie cada afirmação em termos de quanto é PROVÁVEL ou IMPROVÁVEL para esses efeitos como consequências do consumo da cafeína. As possibilidades de respostas são: 1 = Muito improvável; 2 = Improvável; 3 = Um pouco improvável; 4 = Um pouco provável; 5 = Provável; 6 = Muito provável. Baseie suas respostas no produto com cafeína que escolheu. Se você usa muitos tipos de produtos com cafeína, escolha o mais usual para basear suas respostas, ou você pode optar por basear suas respostas em “cafeína/café”.

**Itens**	**Muito Improvável**	**Improvável**	**Um Pouco Improvável**	**Um Pouco Provável**	**Provável**	**Muito Provável**
1. Cafeína/café me dá ânimo quando estou cansado	□	□	□	□	□	□
2. Eu fico extrovertido quando tomo cafeína/café	□	□	□	□	□	□
3. Cafeína/café me ajuda a não comer mais do que deveria	□	□	□	□	□	□
4. Fico facilmente estressado depois de tomar cafeína/café	□	□	□	□	□	□
5. Cafeína/café melhora meu desempenho físico	□	□	□	□	□	□
6. Fico menos cansado depois de tomar cafeína	□	□	□	□	□	□
7. A cafeína/café tira minha fome	□	□	□	□	□	□
8. Fico triste quando não tomo cafeína/café	□	□	□	□	□	□
9. Cafeína/café melhora meu humor	□	□	□	□	□	□
10. Eu fico ansioso quando não tomo cafeína/café	□	□	□	□	□	□
11. Eu me sinto angustiado quando tomo cafeína/café	□	□	□	□	□	□
12. Eu me exercito melhor depois de tomar cafeína/café	□	□	□	□	□	□
13. Eu sinto muita falta de cafeína/café quando não tomo	□	□	□	□	□	□
14. Eu não gosto do jeito que eu me sinto após tomar cafeína/café	□	□	□	□	□	□
15. Eu me sinto mal se ficar sem cafeína/café	□	□	□	□	□	□
16. Cafeína/café aumenta minha motivação para trabalhar	□	□	□	□	□	□
17. Eu me sinto mais confiante depois de tomar cafeína/café	□	□	□	□	□	□
18. Tomar cafeína/café a qualquer hora do dia atrapalha o meu sono	□	□	□	□	□	□
19. Quando tomo cafeína/café fico nervoso(a)	□	□	□	□	□	□
20. Quando tomo cafeína/café fico mais alerta	□	□	□	□	□	□
21. Mesmo quando tomo uma pequena quantidade de cafeína/café fico ansioso	□	□	□	□	□	□
22. Cafeína/café melhora minha concentração	□	□	□	□	□	□
23. Quando tomo cafeína/café fico mais amigável	□	□	□	□	□	□
24. Eu tenho que tomar cafeína/café todos os dias	□	□	□	□	□	□
25. Cafeína/café me faz suar	□	□	□	□	□	□
26. Cafeína/café me faz pular refeições	□	□	□	□	□	□
27. Tenho muita vontade de tomar cafeína/café se não tiver tomado a quantidade de sempre	□	□	□	□	□	□
28. Tomar cafeína/café na hora de dormir atrapalha meu sono	□	□	□	□	□	□
29. Cafeína/café me deixa irritado	□	□	□	□	□	□
30. Eu desejo cafeína/café o tempo todo	□	□	□	□	□	□
31. Cafeína/café me ajuda a trabalhar por mais tempo	□	□	□	□	□	□
32. Cafeína/café me faz sentir feliz	□	□	□	□	□	□
33. Eu não funciono sem tomar cafeína/café	□	□	□	□	□	□
34. Quando tomo cafeína/café meu coração acelera	□	□	□	□	□	□
35. Eu tenho dificuldade em começar o dia sem tomar cafeína/café	□	□	□	□	□	□
36. Sinto dor de estômago quando tomo cafeína/café	□	□	□	□	□	□
37. Eu não conseguiria parar de tomar cafeína/café	□	□	□	□	□	□
38. Tomar cafeína/café no final da tarde atrapalha o meu sono	□	□	□	□	□	□
39. Cafeína/café me ajuda a regular o peso	□	□	□	□	□	□
40. Quanto não tomo cafeína/café sinto dor de cabeça	□	□	□	□	□	□
41. Cafeína/café melhora minha atenção	□	□	□	□	□	□
42. Eu fico mais extrovertido(a) quando tomo cafeína/café	□	□	□	□	□	□
43. Cafeína/café me ajuda a me exercitar por mais tempo	□	□	□	□	□	□
44. Sinto-me mais disposto quando tomo cafeína/café	□	□	□	□	□	□
45. Cafeína/café me faz sentir com mais energia	□	□	□	□	□	□
46. Cafeína/café diminui o meu apetite	□	□	□	□	□	□
47. Tomar cafeína/café no final do dia não me deixa dormir	□	□	□	□	□	□
